# Management of Oligo-teratospermia with Vajikarana Chitkitsa resulting in healthy natural conception: A case report

**DOI:** 10.1016/j.jaim.2025.101190

**Published:** 2025-08-07

**Authors:** Swarda R. Uppin, T.U. Aravinth

**Affiliations:** Department of Rasayana Evam Vajikarana (Ayurveda Preventive and Reproductive Medicine), KAHER's Shri BMK Ayurveda Mahavidyalaya, Shahapur, Belagavi, Karnataka, 590003, India

**Keywords:** Ksheenashukra, Oligo-teratozoospermia, Male infertility, Vajikarana chikitsa, Case report

## Abstract

Oligo-teratozoospermia is a condition affecting the normal sperm count and morphology leading to male factor infertility, majorly resulting due to improper lifestyle adaptations. In Ayurveda literature, the condition is well elaborated a*s Ksheenashukra* having specified treatment modalities. This paper presents a case of infertile couple where in the male was diagnosed with Oligo-teratozoospermia, and was advised for ART due to teratogenecity. The patient approached Ayurveda fertility centre and was subjected to Ayurveda treatment with initial counseling, followed by *Shodhana chikitsa* and 2 courses of *Vajikarana aushadhi* prayoga. Within 3 months, the outcome resulted from Oligo-teratozoospermia to normozoospermia and the partner conceived within 2 cycles. The female experienced a safe and healthy antenatal phase and the couple was blessed with a baby boy.

## 1 Introduction

Infertility is a serious health condition prevailing globally and is known to affect 1 in 6 couples worldwide [1]. About 50 % of infertility is contributed by the male factor, including gametogenic defects [2]. One such defect arising out of abnormal spermatogenesis is Oligo-teratozoospermia. The pathological state is diagnosed by performing a Semen analysis wherein the sperm count is lower than 15 million per ml (Oligozoospermia) and normal sperm morphology lower than 4% (Teratozoospermia) [3]. The spermatogenic deformity mainly arises due to adaptation of inappropriate lifestyle practices or could be genetic in origin. In the western medicine, the management adopted for mild Oligo-teratozoospermia is IUI and moderate to severe Oligo-teratozoospermia is IVF. In either way, ART measures are adopted to get the female partner impregnated [4].

In Ayurveda, the seminal defects (retodushti) and spermatogenic defects (shukra dushti) are well elaborated and an entire branch called Vajikarana Tantra has been dedicated for the enhancement and treatment of the reproductive system. The condition of Oligo-teratozoospermia is a response to the dushti of vata-pitta dosha and is correlated to one of the various presentations of Ksheena shukra [5]. Vajikarana tantra and Shukrajanana gana dravya are a group of drugs indicated to correct the shukra dushti and enhance its production by restoring the reproductive physiology [6]. This paper presents a case of Oligo-teratozoospermia, wherein the couple was suggested to undergo IVF for impregnation. However, the couple approached Ayurveda fertility centre and the male was treated on the principles of Vajikarana Tantra.

## 2 Patient's information

A 29 year male patient along with his 27 year wife visited Ayurveda Fertility Centre with complaint of failure for conception. They were in a non-consanguineous marriage of 3.5 years and the intensity of trying for conception increased since past 2.5 years. They had a healthy sexual life, adequate knowledge of postures, ovulation and conception, and no history of use of contraceptives. The couple was evaluated wherein all the factors investigated for the female were within normal limits.

The male was into IT by profession and had a stressful job with increased electronic usage. He revealed his chronic and habitual consumption of 180ml of 40% alcohol per day, with a history of smoking 5–6 cigarettes per day until last 1 year, which he had now reduced to an occasional smoker. In addition to daily meals, he would consume fast foods at regular intervals of time. In association, he had a history of Gouty arthritis a few years ago and underwent treatment for the same. A month prior to his visit, he was diagnosed as Oligo-teratozoospermia and was advised to opt for in-vitro fertilization owing to the teratogenicity. He was aware that how a history of alcohol and tobacco consumption may affect his fertility, and with much apprehension he visited the Ayurveda fertility centre.

## 3 Clinical findings

The patient was normal, conscious, oriented, and well responding to oral commands, with stable vitals and no associated presentations. Physically, he was well built and had well developed secondary sexual characteristics; his genital examination was normal with respect to appearance and palpation of penis, scrotum and testicles. However, the left testes had mild presentation of a bag of worms on palpation, indicating the presence of mild Varicocele (grade-2).

## 4 Timeline

The timeline of the course of patient care is stated in Table 1.

**Table 1:** Timeline of the course of patient care.

25.01.2023	The couple visited the Ayurveda Fertility Centre and the male was diagnosed as Ksheena shukra (Oligo-teratozoospermia), through Retopariksha, which was contributing to the infertile state of the couple. He was evaluated, counselled and the plan of care was explained.
28.01.2023	Initiated deepana-pachana with *Agnitundi vati* 1 BD
01.02.2023	Snehapana with *Shatavari ghrita*
07.02.2023	Posted for *Virechana karma* followed by samsarjana karma of 5 days
12.02.2023	Post *samsarjana*, first course of *shaman aushadi* was prescribed, as mentioned in Table 2
25.03.2023	Retopariksha was repeated at completion of first course of medications. Based on the improvement, second course of shaman aushadi was prescribed, as mentioned in Table 2
04.05.2023	Retopariksha was repeated towards the completion of second course of medications
16.05.2023	Partner conceived during the second course of medication (LMP 10.04.2023)
09.01.2024	Partner delivered a baby boy through vaginal delivery

## 5 Diagnostic assessment

The patient was advised semen analysis, for its macroscopic and microscopic findings as per updated WHO guidelines [7], and further interpreted on the guidelines stated in the Ayurveda literature [8]. The microscopic analysis of semen revealed the presence of low sperm count, abnormal morphological forms of sperms and a few pus cells. It was observed from the macroscopic analysis, the presence of yellowish white discoloured semen, high pH value and presence of phenilata. These findings signify the involvement of vata-pitta dushti, and thus the *Retopariksha* (Ayurveda interpretation of Semen analysis) was reported as *Ksheena shukra duhsti* (Oligo-teratozoospermia).

## 6 Therapeutic intervention

The patient was educated and counselled for following *nidanaparivarjana* and adapting a better lifestyle. For initial 3 days, *Agnitundi vati* 1 BD was advised to achieve *deepana* and *pachana* effect. Day 4 onwards, *Snehapana* was administered with *Shatavari ghrita* for a period of 4 days with a starting dose of 30ml, followed by 75ml, 100ml and 140ml, until *Samyak Snighda Lakshana* were observed. This was followed by 3 days of *vishrama kala* where *Sarvanga* abhyanga with Pinda taila followed by *bashpa* *sweda* were performed and *pitta vardhaka ahara* was advised. The following day, *Trivrut lehya* 50 g with *Draksha hima* 100ml was administered to achieve *Virechana*.

The patient attained 18 vega of *Madhyama shuddhi* and *Samsarjana krama* was advised for 5 days. On completion of *samsarjana karma*, *shamana aushadhi* were prescribed. Table 1 depicts the timeline of the course of patient care and Table 2 depicts the dose and duration of prescribed courses of *shamana aushadhi*.

**Table 2:** Courses of Shamana aushadhi.

Course 1 of Shamana aushadhi			
Sl no	Drug	Dose	Duration
1	*Kapikacchu beeja churna* with milk	6 gm BD a/c	45 days
2	Tab *Shivagutika*	1 BD	45 days
**Course 2 of shamana aushadhi**			
1	*Chyawanprasha avaleha* with water	6 gm BD a/c	60 days
2	Ta*bShivagutika*	1 BD	60 days
3	*Chandraprabha vati*	2 BD	60 days
4	*Kokilaksha kashayam*	15 ml BD	60 Days

## 7 Follow up & outcomes

Semen analysis was repeated at the completion of the first course of medications. The macroscopic parameters of colour turned to normal and pH reduced indicating improvement. The microscopic parameters of sperm count and normal sperm morphology increased, while pus cells decreased, signifying improvement. Semen analysis was repeated for the second time at the completion of the second course of medications, where in all parameters including phenilata were within the normal range. Table 3 depicts the report of semen analysis, before, during and after treatment. The couple was advised for natural intercourse and the female conceived towards the end of second course of medications. She underwent a vaginal delivery without any complications and the couple was blessed with a healthy baby boy. No adverse events were encountered by the couple in the entire course from diagnosis to delivery.

**Table 3:** Report of Semen analysis at various time points during the treatment.

Parameters	Pre treatment	During treatment	Post treatment
**Abstinence**	3 days	4 days	3 days
**Condition of container**	Sterile	Sterile	Sterile
**Method of collection**	Masturbation	Masturbation	Masturbation
**Time of collection**	12:50 p.m.	10:15 a.m.	02:00 p.m.
**Liquefaction time**	35 min	10 min	10 min
**Appearance**	Opalescent	Opalescent	Opalescent
**Colour**	Yellowish white	Greyish white	Greyish white
**Volume**	2 ml	2 ml	2.5 ml
**pH**	9	8	7.6
**Viscosity**	Absent	Absent	1 cm
**Sperm count**	6 million/ml	41 million/ml	48 million/ml
**Motility:**			
**PR (RLP + SLP)**	54 % (28 + 26)	55 % (41 + 14)	87 % (62 + 25)
**NP**	05 %	20 %	03 %
**IM**	43 %	26 %	10 %
**Agglutination**	Absent	Absent	Absent
**Type**	NA	NA	NA
**Non sperm cells**			
**Pus cells**	6-7/hpf	2-3/hpf	1-2/hpf
**Macrophages**	Nil	Nil	Nil
**Epithelial cells**	Nil	Nil	Nil
**Immature cells**	Nil	Nil	Nil
**Morphology:**			
**Total Normality**	01 %	10 %	32 %
**Head abnormality**	07 %	25 %	16 %
**Mid-piece abnormality**	80 %	15 %	21 %
**Tail abnormality**	12 %	50 %	31 %
**Phenila**	Present	Present	Absent
**Avasadi**	Absent	Absent	Absent

## 8 Discussion

Oligo-teratozoospermia is a condition that is majorly caused due to improper lifestyle practices. The toxins from alcohol and tobacco lead to increased scrotal temperature causing hindrance in spermatogenesis. Further, these factors decreases testosterone production and induce sperm mutation, resulting in decreased sperm count and morphological defects [2]. In addition, the regular consumption of fast foods together vitiate the *vata-pitta dosha* due to its *katu, teekshna, ruksha* and *ushna guna*, particularly at the shukravaha srotas presenting in *Ksheena-shukra dushti* [9]. Thus chikitsa aimed at counseling the patient for nidana parivarjana and *Vajeekarana chikitsa* to achieve *Shukra Prasadana* [10]. As a pre-requisite to *Vajeekarana*, shodhana karma was administered.

Considering the previous history of *vatarakta* and currently *ksheena shukra*, *Shatavari ghrita* was indicated for *snehapana*, which mainly contains *Shatavari*; it is *Madhura rasa* and *Sheeta* in *virya* and is *vata-pitta dosha samana*. Moreover, it also contains drugs such as *gokshura*, *vidarikanda* and *yashtimadhu* etc which is also *madhura rasa* and *vipaka* possessing *rasayana* and *vrushya* properties that alleviate the vitiated *doshas*. *Virechana* with *trivrit lehya* was administered as it is the treatment of choice in *pitta pradhana* conditions and is known to aid in *beeja karmukata* [11].

*Kapikachu beeja churna* is *Madhura* and *tikta* in *rasa*, *brimhana* & *vrushya* by nature and does poshana of rasadi dhatu [12]. A study showed that Mucuna pruriens was found to act on stabilizing Hypothalamo-Pituitary-Gonadal pathway and further enhances sperm production and motility, as it contains mucunine and mucunadine, zinc, magnesium and ascorbic. [13].

*Shilajitu* is the main ingredient of *Shivagutika* which has *Madhura*, *Katu rasa* and *vipaka*; its action of *Vrushya*, *Rasayana* and *Balya* is based on its *yogavahitwa* in a shorter period of time [14]. It contains fulvic acid which is an anti-oxidant and has shown to increase sperm production and improve sexual desire [15]. The other ingredients like *guduchi, bala* and *kakolyadi gana* contribute as anti-oxidant and anti-inflammatory in preventing varicocele condition, which would otherwise lead to spermatogenic defects [16].

Chyawanprash contains amalaki which is *pancha rasa Pradhana* with sheeta *veerya* and *Madhura vipaka*. It is rich in vitamin C and is well known to improve semen quantity and quality. *Chyawanprash* also helps in improving functioning of endocrine system and maintaining hormone secretion. With ingredients like *Aswagandha*, *Gokshura*, *Vidarikanda*, *Bala*, *Jivanti*, *Mashaparni*, *Vamsalochana* are mainly contributed for aphrodisiac qualities [17].

*Kokilaksha kashayam* contains drugs like *kokilaksha* and *guduchi*. *Kokilaksha* is vrushya and is proven to improve spermatogenesis with significant increase in sexual attributes with increased sperm count as well as fructose levels from seminal vesicles. *Guduchi* has anti-inflammatory action and together help in the elimination of excess uric acid, and is indicated in *Vatarakta*. It is *vata-pitta shamaka* and also helps reduce the effect of associated varicocele and prevent recurrence of gouty arthritis [18].

## 9 Conclusion

The Ayurveda literature has well elaborated the sukra dushti and its specific treatment. Administration of the appropriate treatment in the appropriate avastha shall help in better outcome. The case of Oligo-teratozoospermia, which was suggested for ART was well treated with ayurveda principles alone and which significantly resulted from ksheena shukra to prashastha shukra, within a span of 3 months. The partner conceived and experienced a safe and healthy antenatal phase to deliver a healthy male child.

## Informed consent

The informed consent regarding documentation and publication of the case was obtained from the patient.

## Patient perspective

The couple expressed that they were suggested to undergo IVF but wished to have a normal conception. They approached Ayurveda with apprehension but were now overwhelmed about the improvement in the fertility profile and conception through Ayurveda treatment. The husband expressed that through Ayurveda treatment, he had begun to feel enthusiastic and the associated complaints of gout and occasional hyperacidity were cured.

## Author contributions

Conceptualization- SRU; Methodology- SRU; Validation- SRU; Formal analysis- SRU, TUA; Investigation- SRU; Resources- SRU, TUA; Data curation- SRU, TUA; Writing – original draft- TUA; Writing – review and editing- SRU; Visualization- SRU, TUA; Supervision- SRU.

## Declaration of generative AI in scientific writing

None.

## Funding sources

None.

## Conflict of interest

The authors declare that they have no known competing financial interests or personal relationships that could have appeared to influence the work reported in this paper.
